# Engineering building blocks for self-assembling protein nanoparticles

**DOI:** 10.1186/1475-2859-9-101

**Published:** 2010-12-30

**Authors:** Esther Vázquez, Antonio Villaverde

**Affiliations:** 1 Institute for Biotechnology and Biomedicine, Universitat Autònoma de Barcelona, Bellaterra, 08193 Barcelona, Spain; 2 Department of Genetics and Microbiology, Universitat Autònoma de Barcelona, Bellaterra, 08193 Barcelona, Spain; 3 CIBER de Bioingeniería, Biomateriales y Nanomedicina (CIBER-BBN), Bellaterra, 08193 Barcelona, Spain

## Abstract

Like natural viruses, manmade protein cages for drug delivery are to be ideally formed by repetitive subunits with self-assembling properties, mimicking viral functions and molecular organization. Naturally formed nanostructures (such as viruses, flagella or simpler protein oligomers) can be engineered to acquire specific traits of interest in biomedicine, for instance through the addition of cell targeting agents for desired biodistribution and specific delivery of associated drugs. However, fully artificial constructs would be highly desirable regarding finest tuning and adaptation to precise therapeutic purposes. Although engineering of protein assembling is still in its infancy, arising principles and promising strategies of protein manipulation point out the rational construction of nanoscale protein cages as a feasible concept, reachable through conventional recombinant DNA technologies and microbial protein production.

## Commentary

Targeted drug and nucleic acid delivery, in which biodistribution is achieved by ligand-receptor interactions, is a promising way to reduce drug toxicity and enhance effectiveness, what is of special relevance for the treatment of cancer and chronic viral diseases, among others. In gene therapy, the term 'artificial viruses' has been proposed to describe virus-like constructs exhibiting specific viral functions that are relevant to cell recognition, penetration and compartment-aimed release of cargo nucleic acids [1-3]. This nanoparticle-based concept, which can be also extended to chemical drug delivery, involves the use of refillable cages and the incorporation of functional agents for cell receptor binding, cellular uptake and eventually endosomal escape and nuclear delivery. Lipidic and polymeric nanoparticles have been under continuous development during the last four decades [4-6], while nanofibers, nanowires, carbon nanotubes and other types of nanosized cages are being progressively incorporated into the drug delivery scenario [7]. The desired molecular organization, size dispersion and geometry of these constructs is achieved by mechanical and chemical approaches, through fine micro- or nanofabrication procedures [8,9]. Most of these particles are blind; therefore they require to be functionalized with targeting agents for specific cell attachment, being proteins the most efficient, versatile and tunable tools for cell and tissue targeting.

Advances in proteomics and systems biology have permitted to expand the existing catalogues of molecular markers relevant in biomedicine, specially those of interest in cancer diagnosis and therapy [10-14]. In parallel, growing numbers of peptides, antibodies and other protein ligands suitable for cell targeted delivery are becoming available [15-25], supported by the efforts in medical-focused applications of peptide display technologies [20,26-29]. All these findings provide a growing spectrum of therapeutic opportunities in the context of innovative and personalized medicines.

Interestingly, protein-only entities are appealing candidates as building blocks of drug delivery cages [30,31]. Their biocompatibility, biological fabrication, functional diversity and versatility of design though protein engineering (assisted by *in silico *instruments) make them extremely plastic and powerful materials in comparison with liposomes and other types of artificial viruses. Importantly, modular approaches to protein engineering permit the accommodation of several virus-like functions in single (hybrid) polypeptide chains [16,25,32]. In addition, since many microbial and non microbial organisms are being used as cell factories for therapeutic proteins [33], a wide spectrum of biological platforms, molecular tools and comparative expertise in alternative production strategies is available [34]. In particular, bacterial cells are generically good producers of diverse nanomaterials of medical application, including polymers, metal particles and protein particles [35].

The microbial production of protein particles of biomedical interest has been mainly focused on bacteriophages for peptide display, virus-like particles as immunogens and flagella and filamentous phages as templates for micro- and nanofabrication [35,36]. Apart from peptide-displaying filamentous phages, which have been widely explored for targeted delivery of DNA and conjugated drugs in cell culture but also in vivo [37-39], these particles have been in general hardly adaptable to cell-targeted drug delivery.

**Figure 1:**
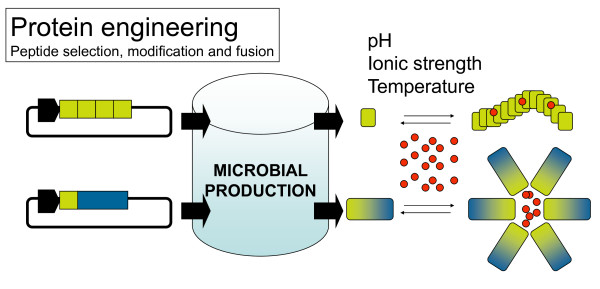
**Self-assembling peptide sequences (yellow boxes) are usually identified from nature, obtained by trial-and-error protein engineering approaches or isolated from combinatorial libraries**. Peptides can be produced in microbial cells as tandem repeats or fused to carrier proteins for further recovery and isolation by proteolysis. Eventually, self-assembling peptides can be produced as fused to multifunctional proteins (blue), to generate self-organizing artificial viruses. Ideally, the assembling of both single peptides and more complex protein building blocks into supramolecular constructs should be controlled in vitro by simple physicochemical parameters, to permit the incorporation of the cargo drug (in red).

Ideally, polypeptides with specific functions relevant to drug delivery (mimicking those of viruses, [16]), should be engineered to self-assemble as nanoparticles with desired nanoscale properties, namely size and geometry, and produced in simple heterologous hosts. The construction of protein particles from their building blocks, as it occurs in natural viruses, can not be achieved by conventional nano-fabrication but through the selection of protein sequences promoting regular protein-protein interactions in absence of unspecific aggregation. In this regard, sets of both natural and non-natural amino acid sequences have been identified that promote peptide self-assembling in diverse patterns. Among them hydrogels, bilayers and nanofibers show important applications in tissue engineering but also in drug delivery [40-44]. Peptide self-assembling can be eventually controlled by pH, temperature and other environment parameters [45-47], and it often involves the formation of amyloid-like, beta-sheet cross-molecular interactions [41] similar to those governing the formation of bacterial inclusion bodies [48-51]. Amyloid-like architecture is probable driving also the assembling of protein fibers formed by misfolding prone recombinant proteins in the cytoplasm of recombinant bacteria [52,53]. Interestingly, some of these short peptides have been successfully produced in bacteria by means of conventional recombinant DNA approaches, fused to carrier proteins or as tandem repeats to enhance their stability [44,54-56] (Figure 1).

Very few of these peptides have been yet incorporated to larger, modular protein constructs for drug delivery to form multifunctional, virus-like protein cages. However, a couple of examples from our own experience prove the feasibility of the concept. In this regard, poly-lysine stretches, commonly used as cationic peptides for DNA condensation [57], confer architectonic properties to engineered beta-galactodidases, which already contained several functional motives for cell receptor binding and nuclear delivery. These chimerical enzymes formed amorphous nanoparticles [58,59], and have been proved to be excellent artificial viruses for in vivo gene delivery to the nervous system in ischemia models and for both histological and functional recovery of injured animals [60,61]. Also, poly-arginine peptides, again used by their DNA condensation properties promoted the self-assembling of scaffold green fluorescent proteins as monodisperse, highly regular 20 nm-nanoparticles useful for DNA and protein delivery [62]. These particles are formed irrespective of the solubility exhibited by the protein under different storage conditions [62,63], proving high regularity and consistence in their architectonic schemes. Interestingly, once exposure to cultured mammalian cells, the intracellular trafficking of these entities is extremely efficient, showing a fast and steady nuclear accumulation in fluorescent forms [62,64]. The sticky nature of cationic peptides, although not completely solved, does not seem to involve cross beta-sheet interactions.

These last results provide a valid proof of concept of the incorporation of self-assembling peptides to complex protein building blocks of artificial viruses (Figure 1), although this is still far from rational design. Moreover, steady conceptual advances in the biology of protein-protein interactions and the easy bioproduction of chimerical proteins in microbial hosts, permit to envisage further progresses in the design of protein cages for drug delivery based on both protein engineering and the exploitation of microbial cell factories.

## Competing interests

The authors declare that they have no competing interests.

## Authors' contributions

EV and AV have equally contributed to this work.
